# Supplementary material for: Genetic risk score in multiple sclerosis is associated with unique gut microbiome
Source: Sci Rep. 2023 Sep 27;13:16269. doi: 10.1038/s41598-023-43217-4 (PMC10533555; doi:10.1038/s41598-023-43217-4)
Supplement: Supplementary file 2 — Supplementary Information 1. [file 41598_2023_43217_MOESM2_ESM.pdf]

Microbiome R markdown

R Markdown

```
library(tidyverse)
library(ggplot2)
library(readxl)
library(ggpubr)
# Alpha indices
#Phylogenetic diversity index
##########################################################
new_pd_faith<-read_excel("H:/CHG/Shukla_Sanjay/Noha/MS Gut Microbiome/16s manuscript with genetic risk s
ggplot(new_pd_faith, aes(x = SMTYPE, y = PD
, fill = SMTYPE)) +  # Manually specified filling color
  geom_boxplot(fatten=3) +
  scale_fill_manual(values = c("royalblue4", "red")) +
  theme(text = element_text(size=20)) +
  geom_jitter(shape=16, position=position_jitter(0.2)) +
  theme(legend.title = element_text(size=20)) +
  theme(legend.text = element_text(size=15))
```

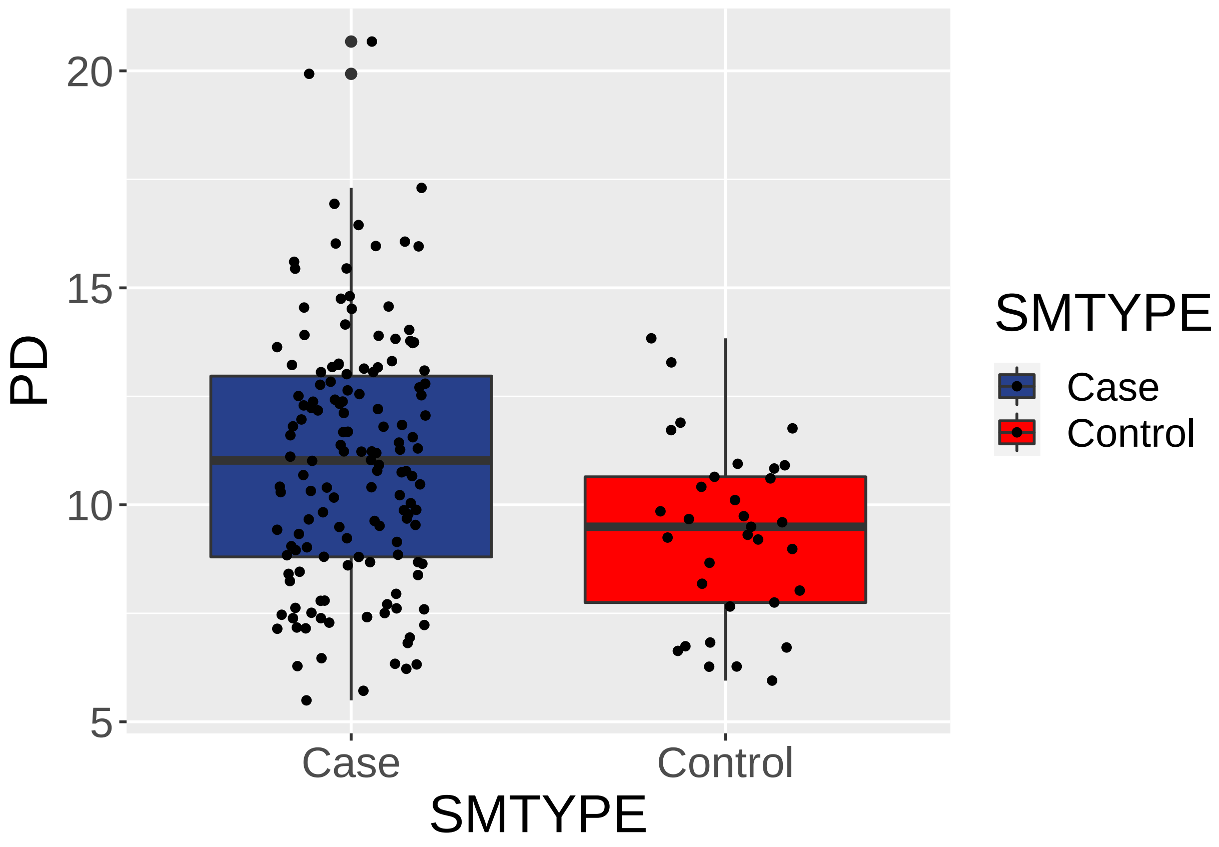


```
# pielou index
##########################################################

Pielou=read.table(file="H:/CHG/Shukla_Sanjay/Noha/MS Gut Microbiome/16s manuscript with genetic risk sco
ggplot(Pielou, aes(x = SMTYPE, y = pielou_e, fill = SMTYPE)) +  # Manually specified filling color
  geom_boxplot(fatten=5) +
  scale_fill_manual(values = c("royalblue4", "red", "purple")) +
  theme(text = element_text(size=20)) +
  geom_jitter(shape=16, position=position_jitter(0.2)) +
  theme(legend.title = element_text(size=20)) +
  theme(legend.text = element_text(size=15))
```

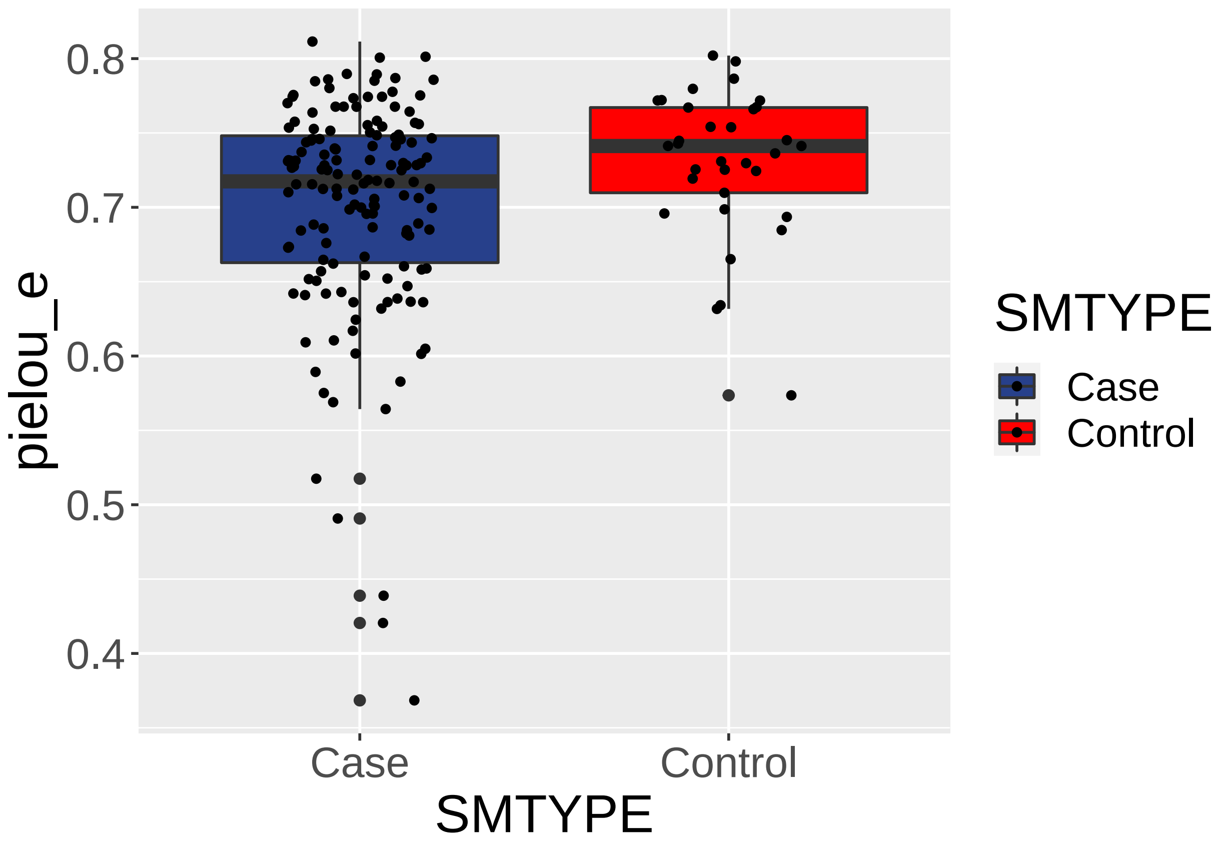


```
###########################################################
# shannon
###########################################################
shannon=read.table(file="M:/Genetic/Projects/Shukla_Microbiome_Seq/results/Robert/qiime2-2019.10/ms/MS_
ggplot(shannon, aes(x = category, y = shannon, fill = category)) +  # Manually specified filling color
  geom_boxplot(fatten=5) +
  scale_fill_manual(values = c("royalblue4", "red", "purple")) +
  theme(text = element_text(size=20)) +
  geom_jitter(shape=16, position=position_jitter(0.2)) +
  theme(legend.title = element_text(size=20)) +
  theme(legend.text = element_text(size=15))
```

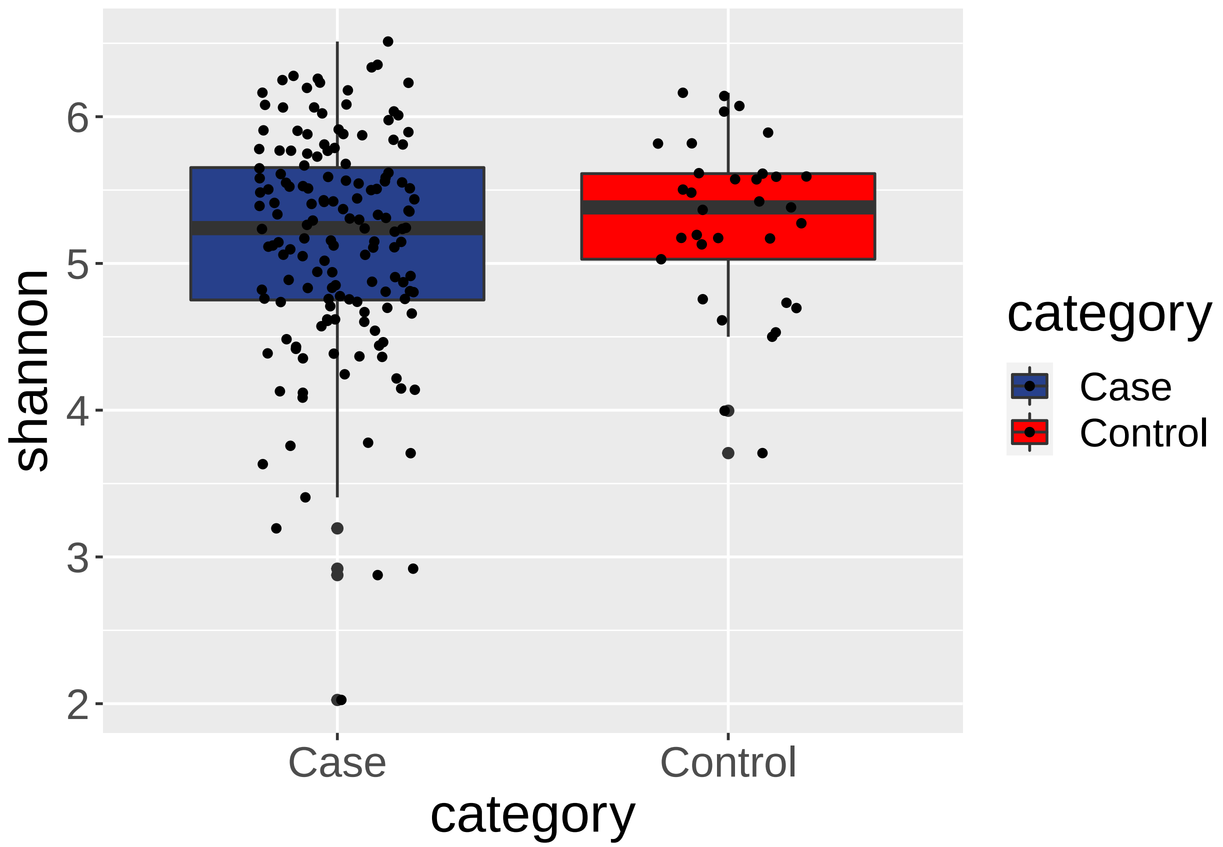


```
# Phylogenetic diversity beween groups 1, group 3, and control
##########################################################
new_pd_faith<-read_excel("H:/CHG/Shukla_Sanjay/Noha/MS Gut Microbiome/16s manuscript with genetic risk s
ggplot(new_pd_faith, aes(x = Group, y = PD , fill = Group)) +  # Manually specified filling color
  geom_boxplot(fatten=3) +
  scale_fill_manual(values = c("RED", "Royal blue", "Medium Blue")) +
  theme(text = element_text(size=20)) +
geom_jitter(shape=16, position=position_jitter(0.2)) +
  theme(legend.title = element_text(size=20)) +
  theme(legend.text = element_text(size=15))
```

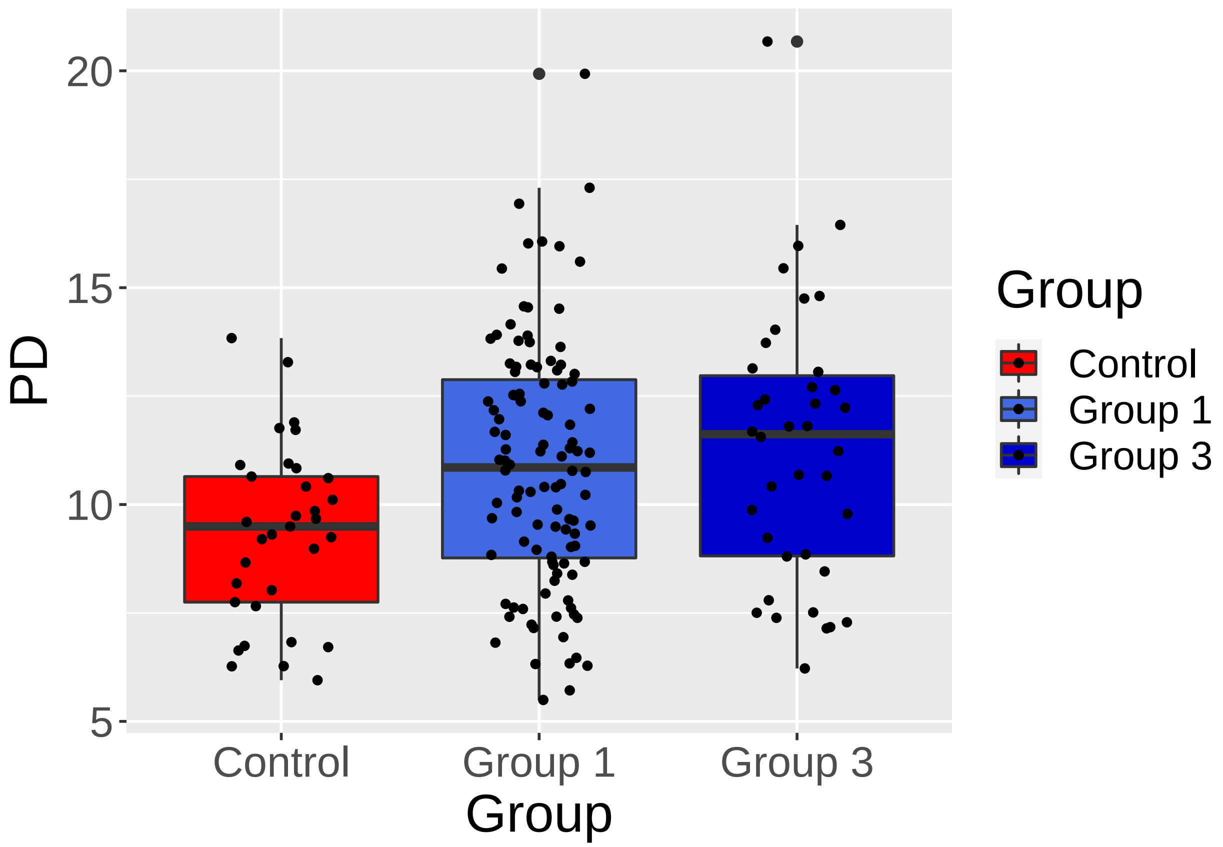


```
library(ggplot2)
library(ggthemes)
library(data.table)
##########################################################
# PCoA
##########################################################
#e.g., qiime tools extract --input-path bray_curtis_pcoa_results.qza --output-path bray_curtis_extracte
datpc=read.table(file="M:/Genetic/Projects/Shukla_Microbiome_Seq/results/Robert/qiime2-2019.10/ms/core-

ggplot(data=datpc, aes(x = PC1_15.10_percent, y = PC2_9.94_percent, color=category)) +
  theme_tufte() +
  geom_point(shape=20, size=6) +

  theme(

    text = element_text(size=35),
    axis.title.x = element_text(margin = margin(t = 15)),
    axis.title.y = element_text(margin = margin(r = 15)),


    legend.title = element_text(size=25),
    legend.text = element_text(size=20),

    panel.border = element_rect(colour = "black", fill=NA, size=2),
    plot.background = element_rect(color = "black", size = 1.5)
```

```
) +
  coord_cartesian(xlim = c(-0.35, 0.40), ylim = c(-0.45, 0.20)) +

scale_color_manual(values=c("A1"="blue", "A3"="orange", "control"="red")) +
geom_point(shape = 1,size = 4,colour = "black")
```

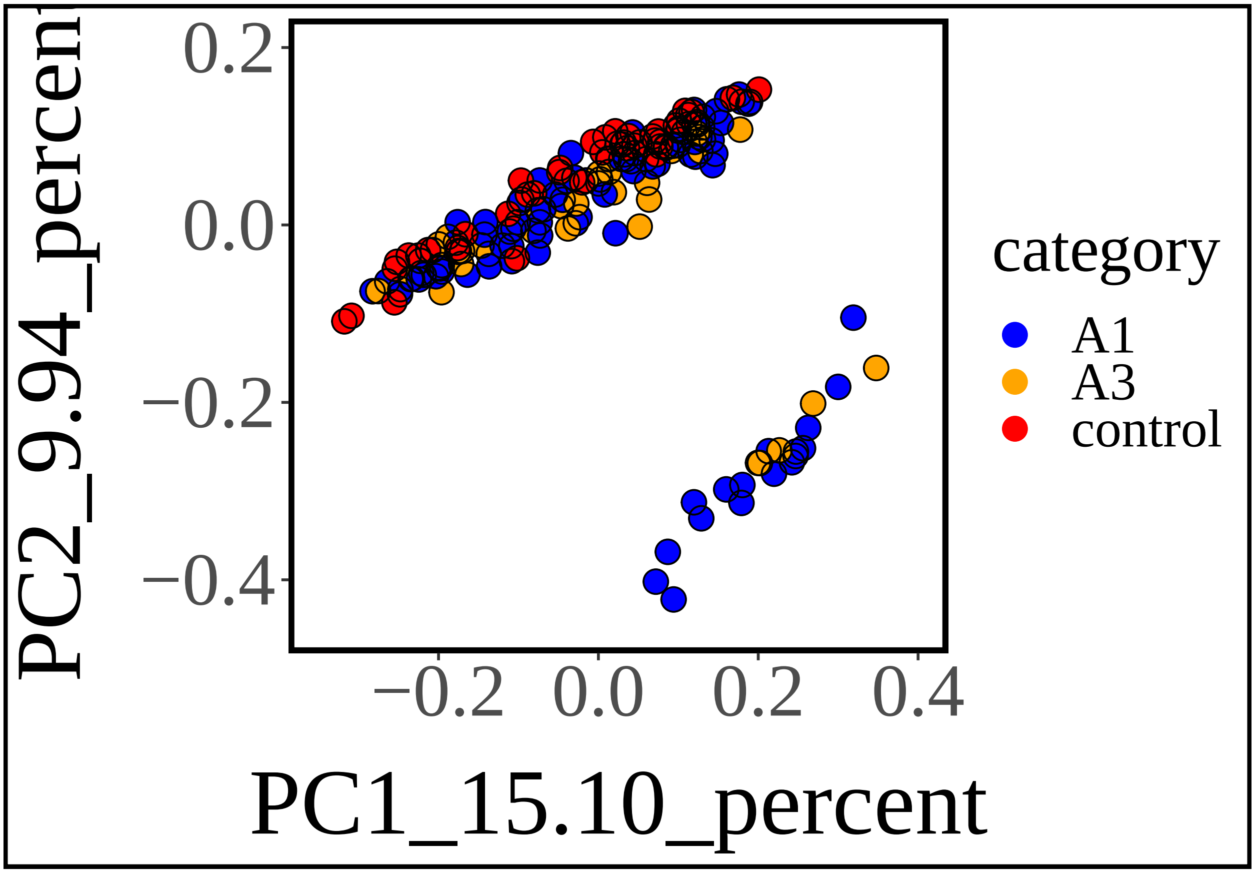


```
##############################################################
# Relative abundance, Phylum
##############################################################
imported_dat=read.csv(file="M:/Genetic/Projects/Shukla_Microbiome_Seq/results/Robert/qiime2-2019.10/ms/
dat=imported_dat

dat2b=list()

#datA=dat2[, grep("A$", colnames(dat2))]
dat2=dat[order(dat$Cluster),]
dat2=dat2[grep("big_cluster", dat2$Cluster),]
#dat3=dat2[, grep("^k__|index", colnames(dat2))]
dat2=dat2[, grep("^k__|index", colnames(dat2))]
dat2=t(dat2)
datAprop=prop.table(dat2, margin=2)
AMpd=rowMeans(datAprop, na.rm=TRUE)
AMpd=as.data.frame(AMpd)
AMpd$otu=rownames(AMpd)
dat2b[[1]]=AMpd
```

```
dat2b[[1]]$Sample = "big_cluster"
colnames(dat2b[[1]])=c("Abundance", "otu", "Sample")

dat2=dat[order(dat$Cluster),]
dat2=dat2[grep("small_cluster", dat2$Cluster),]
dat2=dat2[, grep("^k__|index", colnames(dat2))]
dat2=t(dat2)
datLprop=prop.table(dat2, margin=2)
LMpd=rowMeans(datLprop, na.rm=TRUE)
LMpd=as.data.frame(LMpd)
LMpd$otu=rownames(LMpd)
dat2b[[2]]=LMpd
dat2b[[2]]$Sample = "small_cluster"
colnames(dat2b[[2]])=c("Abundance", "otu", "Sample")

l = list(dat2b[[1]], dat2b[[2]]) #**********************************************
bd=rbindlist(l)

threshold=0.01

bd$otu[bd$Abundance < threshold] = "Rare_combined" #rename low frequency taxa to "Rare_combined"

bd$Sample=as.factor(bd$Sample)
bd$Sample=factor(bd$Sample, levels=c("big_cluster", "small_cluster")) #*********************************
bd$otu=as.factor(bd$otu)

levels(bd$otu)
```

```
## [1] "k__Archaea.p__Euryarchaeota"    "k__Bacteria.p__Actinobacteria"
## [3] "k__Bacteria.p__Bacteroidetes"   "k__Bacteria.p__Firmicutes"
## [5] "k__Bacteria.p__Proteobacteria"  "k__Bacteria.p__Verrucomicrobia"
## [7] "Rare_combined"
```

```
bd$otu=gsub("k__[[:alnum:]]+\\.p__", "", bd$otu)
xt1=xtabs(formula=Abundance~otu+Sample, data=bd)
##########################################################
# Relative abundance, Family
##########################################################
imported_dat=read.csv(file="M:/Genetic/Projects/Shukla_Microbiome_Seq/results/Robert/qiime2-2019.10/ms/
dat=imported_dat

dat2b=list()

dat2=dat[order(dat$Cluster),]
dat2=dat2[grep("big_cluster", dat2$Cluster),]
dat2=dat2[, grep("^k__|index", colnames(dat2))]
dat2=t(dat2)
datAprop=prop.table(dat2, margin=2)
AMpd=rowMeans(datAprop, na.rm=TRUE)
AMpd=as.data.frame(AMpd)
AMpd$otu=rownames(AMpd)
dat2b[[1]]=AMpd
dat2b[[1]]$Sample = "big_cluster"
```

```r
colnames(dat2b[[1]])=c("Abundance", "otu", "Sample")

dat2=dat[order(dat$Cluster),]
dat2=dat2[grep("small_cluster", dat2$Cluster),]
dat2=dat2[, grep("^k__|index", colnames(dat2))]
dat2=t(dat2)
datLprop=prop.table(dat2, margin=2)
LMpd=rowMeans(datLprop, na.rm=TRUE)
LMpd=as.data.frame(LMpd)
LMpd$otu=rownames(LMpd)
dat2b[[2]]=LMpd
dat2b[[2]]$Sample = "small_cluster"
colnames(dat2b[[2]])=c("Abundance", "otu", "Sample")

l = list(dat2b[[1]], dat2b[[2]])
bd=rbindlist(l)

threshold=0.01

bd$otu[bd$Abundance < threshold] = "Rare_combined" #rename low frequency taxa to "Rare_combined"

bd$Sample=as.factor(bd$Sample)
bd$Sample=factor(bd$Sample, levels=c("big_cluster", "small_cluster"))
bd$otu=as.factor(bd$otu)

xt1=xtabs(formula=Abundance~otu+Sample, data=bd)
levels(bd$otu)
```

```
##  [1] "k__Archaea.p__Euryarchaeota.c__Methanobacteria.o__Methanobacteriales.f__Methanobacteriaceae"
##  [2] "k__Bacteria.p__Actinobacteria.c__Actinobacteria.o__Bifidobacteriales.f__Bifidobacteriaceae"
##  [3] "k__Bacteria.p__Actinobacteria.c__Coriobacteriia.o__Coriobacteriales.f__Coriobacteriaceae"
##  [4] "k__Bacteria.p__Bacteroidetes.c__Bacteroidia.o__Bacteroidales.f__Bacteroidaceae"
##  [5] "k__Bacteria.p__Bacteroidetes.c__Bacteroidia.o__Bacteroidales.f__Prevotellaceae"
##  [6] "k__Bacteria.p__Bacteroidetes.c__Bacteroidia.o__Bacteroidales.f__Rikenellaceae"
##  [7] "k__Bacteria.p__Firmicutes.c__Bacilli.o__Lactobacillales.f__Streptococcaceae"
##  [8] "k__Bacteria.p__Firmicutes.c__Clostridia.o__Clostridiales.f__"
##  [9] "k__Bacteria.p__Firmicutes.c__Clostridia.o__Clostridiales.f__Clostridiaceae"
## [10] "k__Bacteria.p__Firmicutes.c__Clostridia.o__Clostridiales.f__Lachnospiraceae"
## [11] "k__Bacteria.p__Firmicutes.c__Clostridia.o__Clostridiales.f__Peptostreptococcaceae"
## [12] "k__Bacteria.p__Firmicutes.c__Clostridia.o__Clostridiales.f__Ruminococcaceae"
## [13] "k__Bacteria.p__Firmicutes.c__Clostridia.o__Clostridiales.f__Veillonellaceae"
## [14] "k__Bacteria.p__Firmicutes.c__Erysipelotrichi.o__Erysipelotrichales.f__Erysipelotrichaceae"
## [15] "k__Bacteria.p__Proteobacteria.c__Gammaproteobacteria.o__Enterobacteriales.f__Enterobacteriacea
## [16] "k__Bacteria.p__Verrucomicrobia.c__Verrucomicrobiae.o__Verrucomicrobiales.f__Verrucomicrobiacea
## [17] "Rare_combined"
```

```r
bd$otu=gsub("k__[[:alnum:]]+\\.p__[[:alnum:]]+\\.c__[[:alnum:]]+\\.o__[[:alnum:]]+\\.f__", "", bd$otu)
bd1=bd[bd==""]<-"Other"
xt2=xtabs(formula=Abundance~otu+Sample, data=bd)
bd$otu=gsub("\\.", "", bd$otu)
###########################################################
# Relative abundance, Genus
###########################################################
```

```
imported_dat=read.csv(file="M:/Genetic/Projects/Shukla_Microbiome_Seq/results/Robert/qiime2-2019.10/ms/
dat=imported_dat

dat2b=list()

dat2=dat[order(dat$Cluster),]
dat2=dat2[grep("big_cluster", dat2$Cluster),]
dat2=dat2[, grep("^k__|index", colnames(dat2))]
dat2=t(dat2)
datAprop=prop.table(dat2, margin=2)
AMpd=rowMeans(datAprop, na.rm=TRUE)
AMpd=as.data.frame(AMpd)
AMpd$otu=rownames(AMpd)
dat2b[[1]]=AMpd
dat2b[[1]]$Sample = "big_cluster"
colnames(dat2b[[1]])=c("Abundance", "otu", "Sample")


dat2=dat[order(dat$Cluster),]
dat2=dat2[grep("small_cluster", dat2$Cluster),]
dat2=dat2[, grep("^k__|index", colnames(dat2))]
dat2=t(dat2)
datLprop=prop.table(dat2, margin=2)
LMpd=rowMeans(datLprop, na.rm=TRUE)
LMpd=as.data.frame(LMpd)
LMpd$otu=rownames(LMpd)
dat2b[[2]]=LMpd
dat2b[[2]]$Sample = "small_cluster"
colnames(dat2b[[2]])=c("Abundance", "otu", "Sample")

l = list(dat2b[[1]], dat2b[[2]])
bd=rbindlist(l)

threshold=0.01

bd$otu[bd$Abundance < threshold] = "Rare_combined" #rename low frequency taxa to "Rare_combined"

bd$Sample=as.factor(bd$Sample)
bd$Sample=factor(bd$Sample, levels=c("big_cluster", "small_cluster"))
bd$otu=as.factor(bd$otu)

xt1=xtabs(formula=Abundance~otu+Sample, data=bd)
levels(bd$otu)
```

```
##  [1] "k__Archaea.p__Euryarchaeota.c__Methanobacteria.o__Methanobacteriales.f__Methanobacteriaceae.g__
##  [2] "k__Bacteria.p__Actinobacteria.c__Actinobacteria.o__Bifidobacteriales.f__Bifidobacteriaceae.g__
##  [3] "k__Bacteria.p__Actinobacteria.c__Coriobacteriia.o__Coriobacteriales.f__Coriobacteriaceae.g__Co
##  [4] "k__Bacteria.p__Bacteroidetes.c__Bacteroidia.o__Bacteroidales.f__Bacteroidaceae.g__Bacteroides"
##  [5] "k__Bacteria.p__Bacteroidetes.c__Bacteroidia.o__Bacteroidales.f__Prevotellaceae.g__Prevotella"
##  [6] "k__Bacteria.p__Bacteroidetes.c__Bacteroidia.o__Bacteroidales.f__Rikenellaceae.g__Alistipes"
##  [7] "k__Bacteria.p__Firmicutes.c__Bacilli.o__Lactobacillales.f__Streptococcaceae.g__Streptococcus"
##  [8] "k__Bacteria.p__Firmicutes.c__Clostridia.o__Clostridiales.f__.g__"
##  [9] "k__Bacteria.p__Firmicutes.c__Clostridia.o__Clostridiales.f__Clostridiaceae.g__Clostridium"
```

```
## [10] "k__Bacteria.p__Firmicutes.c__Clostridia.o__Clostridiales.f__Lachnospiraceae.__"
## [11] "k__Bacteria.p__Firmicutes.c__Clostridia.o__Clostridiales.f__Lachnospiraceae.g__"
## [12] "k__Bacteria.p__Firmicutes.c__Clostridia.o__Clostridiales.f__Lachnospiraceae.g__.Ruminococcus."
## [13] "k__Bacteria.p__Firmicutes.c__Clostridia.o__Clostridiales.f__Lachnospiraceae.g__Blautia"
## [14] "k__Bacteria.p__Firmicutes.c__Clostridia.o__Clostridiales.f__Lachnospiraceae.g__Coprococcus"
## [15] "k__Bacteria.p__Firmicutes.c__Clostridia.o__Clostridiales.f__Lachnospiraceae.g__Dorea"
## [16] "k__Bacteria.p__Firmicutes.c__Clostridia.o__Clostridiales.f__Lachnospiraceae.g__Roseburia"
## [17] "k__Bacteria.p__Firmicutes.c__Clostridia.o__Clostridiales.f__Peptostreptococcaceae.g__"
## [18] "k__Bacteria.p__Firmicutes.c__Clostridia.o__Clostridiales.f__Ruminococcaceae.g__"
## [19] "k__Bacteria.p__Firmicutes.c__Clostridia.o__Clostridiales.f__Ruminococcaceae.g__Faecalibacteriu
## [20] "k__Bacteria.p__Firmicutes.c__Clostridia.o__Clostridiales.f__Ruminococcaceae.g__Oscillospira"
## [21] "k__Bacteria.p__Firmicutes.c__Clostridia.o__Clostridiales.f__Ruminococcaceae.g__Ruminococcus"
## [22] "k__Bacteria.p__Firmicutes.c__Clostridia.o__Clostridiales.f__Veillonellaceae.g__Megasphaera"
## [23] "k__Bacteria.p__Firmicutes.c__Clostridia.o__Clostridiales.f__Veillonellaceae.g__Phascolarctobac
## [24] "k__Bacteria.p__Firmicutes.c__Erysipelotrichi.o__Erysipelotrichales.f__Erysipelotrichaceae.g__"
## [25] "k__Bacteria.p__Proteobacteria.c__Gammaproteobacteria.o__Enterobacteriales.f__Enterobacteriacea
## [26] "k__Bacteria.p__Verrucomicrobia.c__Verrucomicrobiae.o__Verrucomicrobiales.f__Verrucomicrobiacea
## [27] "Rare_combined"
```

```
bd$otu=gsub("k__[[:alnum:]]+\\.p__[[:alnum:]]+\\.c__[[:alnum:]]+\\.o__[[:alnum:]]+\\.f__", "", bd$otu)
bd$otu=gsub("\\.{2}", ".", bd$otu)
bd$otu=gsub("g__\\.", "g__", bd$otu)
bd$otu=gsub("\\.__", "\\.g__", bd$otu)
bd$otu=gsub("[[:alpha:]]+\\.g__", "\\.g__", bd$otu)
bd$otu=gsub("\\.", "", bd$otu)

bd$otu=gsub("g__$", "Other", bd$otu)
bd$otu=gsub("g__", "", bd$otu)
xt2=xtabs(formula=Abundance~otu+Sample, data=bd)

#########################################################
# The Quasi-Conditional Association Test using General Estimating Equations (QCAT-GEE)
#########################################################
library(miLineage)
library(readxl)
otu_1_num_new <- read_excel("//mcrf1/MMRPublic/CHG/Shukla_Sanjay/Noha/MS Gut Microbiome/16s manuscript w
Taxa <- read_excel("//mcrf1/MMRPublic/CHG/Shukla_Sanjay/Noha/MS Gut Microbiome/16s manuscript with gene
cluster_NUM_new <- read_excel("//mcrf1/MMRPublic/CHG/Shukla_Sanjay/Noha/MS Gut Microbiome/16s manuscrip
write.table(otu_1_num_new, file="otu1.txt")
write.table(Taxa, file="tax2.txt")
write.table(cluster_NUM_new,file="sam3.txt")


otu1.temp=read.table("otu1.txt")
tax2.temp=read.table("tax2.txt")
sam3.temp=read.table("sam3.txt")
# make the rownames and coloumn names of both taxa and otu tables
otu.temp.c <- otu1.temp[,-1]
rownames(otu.temp.c) <- otu1.temp [,1]

TAXA.temp.c <- tax2.temp[,-1]
rownames(TAXA.temp.c) <- tax2.temp [,1]
#match the two tables
otu1.reorde.nr = otu1.temp[,match( colnames(otu.temp.c), rownames(TAXA.temp.c ))]
```

```r
#try to remove the column 1 again
OTU.N <- otu1.reorde.nr[,-1]
rownames(OTU.N ) <- otu1.reorde.nr [,1]
#changing files into matrix
sam3.temp.m=as.matrix(sam3.temp)
tax2.temp.m=as.matrix (tax2.temp)
OTU.N.M= as.matrix(OTU.N)
Taxa.temp.c.m=as.matrix(TAXA.temp.c)
QCAT_GEE(OTU.N.M, sam3.temp.m, 1, sam3.temp.m, 1, Taxa.temp.c.m, n.resample = 1000)
```

```
## $lineage.pval
## $lineage.pval$`Two-Part`
##             ;f__Lachnospiraceae; f__[Mogibacteriaceae] f__[Paraprevotellaceae];
## Asymptotic            0.2840303             0.4475827                 0.4388304
## Resampling            0.2178218             0.6039604                 0.2574257
##             f__[Tissierellaceae] f__[Weeksellaceae]; f__Actinomycetaceae;
## Asymptotic           0.13243551          0.7294255           0.1306931
## Resampling           0.05694306          0.5643564           0.3069307
##             f__Bifidobacteriaceae; f__Christensenellaceae; f__Clostridiaceae;
## Asymptotic              0.6090512              0.8529113          0.6936501
## Resampling              0.6336634              0.8910891          0.8019802
##             f__Coriobacteriaceae; f__Dehalobacteriaceae; f__Desulfovibrionaceae
## Asymptotic             0.6958833              0.3725796              0.4425977
## Resampling             0.7128713              0.4752475              0.4356436
##             f__Dethiosulfovibrionaceae f__Enterobacteriaceae;
## Asymptotic                  0.0000000             1.0000000
## Resampling                  0.4554455             0.8316832
##             f__Enterococcaceae; f__Erysipelotrichaceae;_ f__Fusobacteriaceae;
## Asymptotic           0.3327186              0.04637918          0.05367291
## Resampling           0.1584158              0.03496503          0.27722772
##             f__Lachnospiraceae; f__Lactobacillaceae; f__Leuconostocaceae;
## Asymptotic          0.03263683          0.07353704            1.0000000
## Resampling          0.01998002          0.17821782            0.6138614
##             f__Methanobacteriaceae; f__Micrococcaceae; f__mitochondria;__
## Asymptotic               0.1246455          0.5873185          0.5018789
## Resampling               0.1980198          0.6831683          0.4752475
##             f__Moraxellaceae f__Pasteurellaceae f__Peptococcaceae
## Asymptotic        0.5870769          0.4034840         0.7458692
## Resampling        0.5049505          0.4158416         0.5643564
##             f__Peptostreptococcaceae f__Porphyromonadaceae; f__Rikenellaceae;
## Asymptotic                0.1471925           0.000000000         0.3152025
## Resampling                0.4653465           0.008991009         0.4554455
##             f__Ruminococcaceae f__Streptococcaceae; f__Synergistaceae
## Asymptotic          0.1357610            0.8624156         1.0000000
## Resampling          0.1683168            0.9504950         0.8811881
##             f__Veillonellaceae;_ o__Actinomycetales o__Actinomycetales;
## Asymptotic           0.09364934          0.2756097           0.2523611
## Resampling           0.11388611          0.6435644           0.2673267
##             o__Bacteroidales o__Bacteroidales; o__Burkholderiales
## Asymptotic        0.5722187         0.5102790          0.4088249
## Resampling        0.5544554         0.4158416          0.4653465
##             o__Campylobacterales o__Clostridiales o__Clostridiales;
## Asymptotic            0.2599374        0.1608367         0.1898919
```

```
## Resampling            0.3663366        0.1881188         0.2079208
##            o__Lactobacillales; o__Pseudomonadales  o__RB41; o__Synergistales
## Asymptotic          0.06968758          0.4186928 0.6119858       0.08590295
## Resampling          0.03096903          0.1782178 0.6336634       0.28712871
##            ;c__Actinobacteria; ;c__Bacteroidia; c__[Chloracidobacteria];
## Asymptotic           0.4080308       0.02785103               0.6028992
## Resampling           0.4752475       0.06193806               0.7524752
##            c__Actinobacteria; c__Alphaproteobacteria c__Bacilli;
## Asymptotic          0.7487855              0.9004887   0.1411682
## Resampling          0.7623762              1.0000000   0.2574257
##            c__Bacteroidia; c__Betaproteobacteria c__Clostridia;
## Asymptotic       0.7680318             0.5625582      0.9380504
## Resampling       0.7722772             0.3168317      0.9702970
##            c__Gammaproteobacteria c__Methanobacteria; c__Mollicutes
## Asymptotic              0.4708914           0.3043650     0.7889646
## Resampling              0.3663366           0.5346535     0.7128713
##            ;p__Actinobacteria; ;p__Bacteroidetes; p__Acidobacteria;
## Asymptotic           0.2623209          0.5834591         0.6028992
## Resampling           0.3960396          0.5148515         0.6831683
##            p__Actinobacteria p__Actinobacteria; p__Bacteroidetes;
## Asymptotic         1.0000000          0.5971405         0.8239869
## Resampling         0.6831683          0.6138614         0.6732673
##            p__Cyanobacteria; p__Firmicutes p__Firmicutes; p__Proteobacteria
## Asymptotic         0.1439810     1.0000000      0.3087289         0.7056448
## Resampling         0.1782178     0.9405941      0.1584158         0.8514851
##            p__Tenericutes p__Verrucomicrobia k__Archaea; k__Bacteria
## Asymptotic      0.3829985          1.0000000   1.0000000   0.6554995
## Resampling      0.3069307          0.7623762   0.9207921   0.6930693
##            k__Bacteria; k__Bacteria;;
## Asymptotic    0.9281516     0.4777949
## Resampling    0.8415842     0.2772277
##
## $lineage.pval$`Zero-Part`
##            ;f__Lachnospiraceae; f__[Mogibacteriaceae] f__[Paraprevotellaceae];
## Asymptotic           0.09382132             0.5384705               0.1964610
## Resampling           0.07920792             0.5742574               0.2673267
##            f__[Tissierellaceae] f__[Weeksellaceae]; f__Actinomycetaceae;
## Asymptotic           0.09538992          0.4220440           0.1005584
## Resampling           0.10389610          0.5643564           0.1485149
##            f__Bifidobacteriaceae; f__Christensenellaceae; f__Clostridiaceae;
## Asymptotic              0.4365587              0.7977349         0.5415020
## Resampling              0.5643564              0.7920792         0.7425743
##            f__Coriobacteriaceae; f__Dehalobacteriaceae; f__Desulfovibrionaceae
## Asymptotic             0.5567653             0.8347001              0.5216133
## Resampling             0.6237624             0.8118812              0.5544554
##            f__Dethiosulfovibrionaceae f__Enterobacteriaceae;
## Asymptotic                 0.08124916             0.5795377
## Resampling                 0.27722772             0.8613861
##            f__Enterococcaceae; f__Erysipelotrichaceae;_ f__Fusobacteriaceae;
## Asymptotic           0.1818368              0.03763249          0.04850968
## Resampling           0.1584158              0.04495504          0.30693069
##            f__Lachnospiraceae; f__Lactobacillaceae; f__Leuconostocaceae;
## Asymptotic          0.13198720           0.1446784          0.00524896
## Resampling          0.06793207           0.2376238          0.01980198
```

```
##            f__Methanobacteriaceae; f__Micrococcaceae; f__mitochondria;__
## Asymptotic              0.1088254          0.3812148          0.2272260
## Resampling              0.1485149          0.6831683          0.4752475
##            f__Moraxellaceae f__Pasteurellaceae f__Peptococcaceae
## Asymptotic        0.3809968          0.3679662         0.3682156
## Resampling        0.5049505          0.4158416         0.5643564
##            f__Peptostreptococcaceae f__Porphyromonadaceae; f__Rikenellaceae;
## Asymptotic                0.2077852              0.5921457         0.2664025
## Resampling                0.5049505              0.7952048         0.5445545
##            f__Ruminococcaceae f__Streptococcaceae; f__Synergistaceae
## Asymptotic         0.08871899            0.7549972         0.2442499
## Resampling         0.06930693            0.9009901         0.3861386
##            f__Veillonellaceae;_ o__Actinomycetales o__Actinomycetales;
## Asymptotic           0.01915199          0.3915299           0.1496140
## Resampling           0.01398601          0.6435644           0.2079208
##            o__Bacteroidales o__Bacteroidales; o__Burkholderiales
## Asymptotic        0.5012998         0.4667090          0.3714836
## Resampling        0.4950495         0.5148515          0.4851485
##            o__Campylobacterales o__Clostridiales o__Clostridiales;
## Asymptotic            0.1343794       0.09519889         0.3062991
## Resampling            0.3663366       0.11881188         0.4356436
##            o__Lactobacillales; o__Pseudomonadales  o__RB41; o__Synergistales
## Asymptotic          0.02411998          0.2430058 0.6119858       0.04507757
## Resampling          0.02097902          0.1782178 0.6336634       0.26732673
##            ;c__Actinobacteria; ;c__Bacteroidia; c__[Chloracidobacteria];
## Asymptotic           0.3969771       0.4674793                0.3954117
## Resampling           0.3861386       0.4955045                0.7524752
##            c__Actinobacteria; c__Alphaproteobacteria c__Bacilli;
## Asymptotic          0.5436705              0.8478623   0.1553492
## Resampling          0.5940594              0.9900990   0.2277228
##            c__Bacteroidia; c__Betaproteobacteria c__Clostridia;
## Asymptotic       0.4765253             0.3592028      0.9621699
## Resampling       0.5544554             0.3168317      0.9702970
##            c__Gammaproteobacteria c__Methanobacteria; c__Mollicutes
## Asymptotic              0.3997031           0.2982684     0.5913214
## Resampling              0.4851485           0.5346535     0.7128713
##            ;p__Actinobacteria; ;p__Bacteroidetes; p__Acidobacteria;
## Asymptotic           0.9347141          0.3777401         0.3954117
## Resampling           0.9702970          0.4158416         0.7524752
##            p__Actinobacteria p__Actinobacteria; p__Bacteroidetes;
## Asymptotic         0.4162703          0.5705461         0.6043117
## Resampling         0.7029703          0.6633663         0.6732673
##            p__Cyanobacteria; p__Firmicutes p__Firmicutes; p__Proteobacteria
## Asymptotic         0.8384537     0.2823868      0.4237811         0.7265336
## Resampling         0.8811881     0.5742574      0.2574257         0.8514851
##            p__Tenericutes p__Verrucomicrobia k__Archaea; k__Bacteria
## Asymptotic      0.3096443          0.1236054  0.08040973   0.6520826
## Resampling      0.3069307          0.1485149  0.23762376   0.8514851
##            k__Bacteria; k__Bacteria;;
## Asymptotic    0.7454013     0.2884922
## Resampling    0.7920792     0.2772277
##
## $lineage.pval$`Positive-Part`
##            ;f__Lachnospiraceae; f__[Mogibacteriaceae] f__[Paraprevotellaceae];
```

```
## Asymptotic           0.6839922             0.2335740                0.8012727
## Resampling           0.5544554             0.3465347                0.3564356
##            f__[Tissierellaceae] f__[Weeksellaceae]; f__Actinomycetaceae;
## Asymptotic           0.3666457                    1           0.3226310
## Resampling           0.1188811                    1           0.9009901
##            f__Bifidobacteriaceae; f__Christensenellaceae; f__Clostridiaceae;
## Asymptotic             0.6560446              0.5635300         0.6691836
## Resampling             0.6237624              0.7623762         0.7920792
##            f__Coriobacteriaceae; f__Dehalobacteriaceae; f__Desulfovibrionaceae
## Asymptotic            0.6646447            0.09635896              0.2818467
## Resampling            0.6039604            0.06930693              0.2673267
##            f__Dethiosulfovibrionaceae f__Enterobacteriaceae;
## Asymptotic                  0.0000000             1.0000000
## Resampling                  0.4554455             0.8316832
##            f__Enterococcaceae; f__Erysipelotrichaceae;_ f__Fusobacteriaceae;
## Asymptotic                  1               0.2581587           0.2052771
## Resampling                  1               0.2517483           0.4950495
##            f__Lachnospiraceae; f__Lactobacillaceae; f__Leuconostocaceae;
## Asymptotic         0.02996443           0.09731883           1.0000000
## Resampling         0.02697303           0.21782178           0.6138614
##            f__Methanobacteriaceae; f__Micrococcaceae; f__mitochondria;__
## Asymptotic              0.2524055                  1                 1
## Resampling              0.6831683                  1                 1
##            f__Moraxellaceae f__Pasteurellaceae f__Peptococcaceae
## Asymptotic                1         0.37812129                 1
## Resampling                1         0.03960396                 1
##            f__Peptostreptococcaceae f__Porphyromonadaceae; f__Rikenellaceae;
## Asymptotic                0.1775988            0.000000000        0.37642634
## Resampling                0.3762376            0.008991009        0.08910891
##            f__Ruminococcaceae f__Streptococcaceae; f__Synergistaceae
## Asymptotic         0.3891710            0.7007626         1.0000000
## Resampling         0.3762376            0.7623762         0.8811881
##            f__Veillonellaceae;_ o__Actinomycetales o__Actinomycetales;
## Asymptotic           0.7467729          0.1576402           0.5200924
## Resampling           0.9100899          0.2772277           0.6237624
##            o__Bacteroidales o__Bacteroidales; o__Burkholderiales
## Asymptotic        0.4311201         0.4926418          0.4022910
## Resampling        0.4158416         0.3465347          0.2079208
##            o__Campylobacterales o__Clostridiales o__Clostridiales;
## Asymptotic                    1        0.4248456         0.1826122
## Resampling                    1        0.3762376         0.1584158
##            o__Lactobacillales; o__Pseudomonadales o__RB41; o__Synergistales
## Asymptotic          0.5033217                  1       NA        0.5278308
## Resampling          0.3736264                  1       NA        0.9306931
##            ;c__Actinobacteria; ;c__Bacteroidia; c__[Chloracidobacteria];
## Asymptotic          0.3500689      0.005868675                        1
## Resampling          0.5445545      0.001998002                        1
##            c__Actinobacteria; c__Alphaproteobacteria c__Bacilli;
## Asymptotic         0.7627721              0.6941734   0.2329870
## Resampling         0.8712871              0.3168317   0.3168317
##            c__Bacteroidia; c__Betaproteobacteria c__Clostridia;
## Asymptotic       0.8846606                     1      0.8153317
## Resampling       0.8811881                     1      0.7821782
##            c__Gammaproteobacteria c__Methanobacteria; c__Mollicutes
```

```
## Asymptotic              0.4766181           0.2713594             1
## Resampling              0.2178218           0.1485149             1
##            ;p__Actinobacteria; ;p__Bacteroidetes; p__Acidobacteria;
## Asymptotic          0.04953742          1.0000000         1.0000000
## Resampling          0.03960396          0.7722772         0.8118812
##            p__Actinobacteria p__Actinobacteria; p__Bacteroidetes;
## Asymptotic         1.0000000          0.4588075         0.8283447
## Resampling         0.6831683          0.3168317         0.6732673
##            p__Cyanobacteria; p__Firmicutes p__Firmicutes; p__Proteobacteria
## Asymptotic        0.02448596     1.0000000      0.2415287         0.4859569
## Resampling        0.00990099     0.9405941      0.1485149         0.5247525
##            p__Tenericutes p__Verrucomicrobia k__Archaea; k__Bacteria
## Asymptotic      0.3988404          1.0000000   1.0000000   0.4977120
## Resampling      0.6138614          0.7623762   0.9207921   0.4356436
##            k__Bacteria; k__Bacteria;;
## Asymptotic    0.9037902             1
## Resampling    0.7524752             1
##
##
## $sig.lineage
## $sig.lineage$`Two-Part`
## character(0)
##
## $sig.lineage$`Zero-Part`
## character(0)
##
## $sig.lineage$`Positive-Part`
## character(0)
##
##
## $global.pval
##      Simes_Two-Part     Simes_Zero-Part Simes_Positive-Part
##           0.6381119           0.5104895           0.1438561
```
