# Supplementary material for: Genetic risk score in multiple sclerosis is associated with unique gut microbiome
Source: Sci Rep. 2023 Sep 27;13:16269. doi: 10.1038/s41598-023-43217-4 (PMC10533555; doi:10.1038/s41598-023-43217-4)
Supplement: Supplementary file 3 — Supplementary Information 2. [file 41598_2023_43217_MOESM3_ESM.pdf]

Genetic risk

R Markdown

This is an R Markdown document. Markdown is a simple formatting syntax for authoring HTML, PDF, and MS Word documents. For more details on using R Markdown see http://rmarkdown.rstudio.com.

When you click the **Knit** button a document will be generated that includes both content as well as the output of any embedded R code chunks within the document. You can embed an R code chunk like this:

```
library(ggplot2)
library(ggthemes)
library(data.table)
library(readxl)
standard_grs_all_same_mean_UPDATED <-read_excel("N:/Multiple sclerosis/16s paper/grs/standard_grs_all_sa
ggplot(standard_grs_all_same_mean_UPDATED, aes(x = GRS
)) +

  geom_histogram (aes(color = cateogry
, fill = cateogry
),
                 position = "identity", bins = 30, alpha = 0.4) +

  scale_color_manual(values = c("royalblue4", "red")) +
  scale_fill_manual(values = c("royalblue4", "red")) +

  theme(
    text = element_text(size=25),
    legend.title = element_blank(),
    legend.text = element_text(size=25),
    legend.position="top",

    axis.title.x = element_text(margin = margin(t = 15)),
    axis.title.y = element_text(margin = margin(r = 15)),

  )+
  xlab("Polygenic risk score")+
  ylab("Subject count")
```

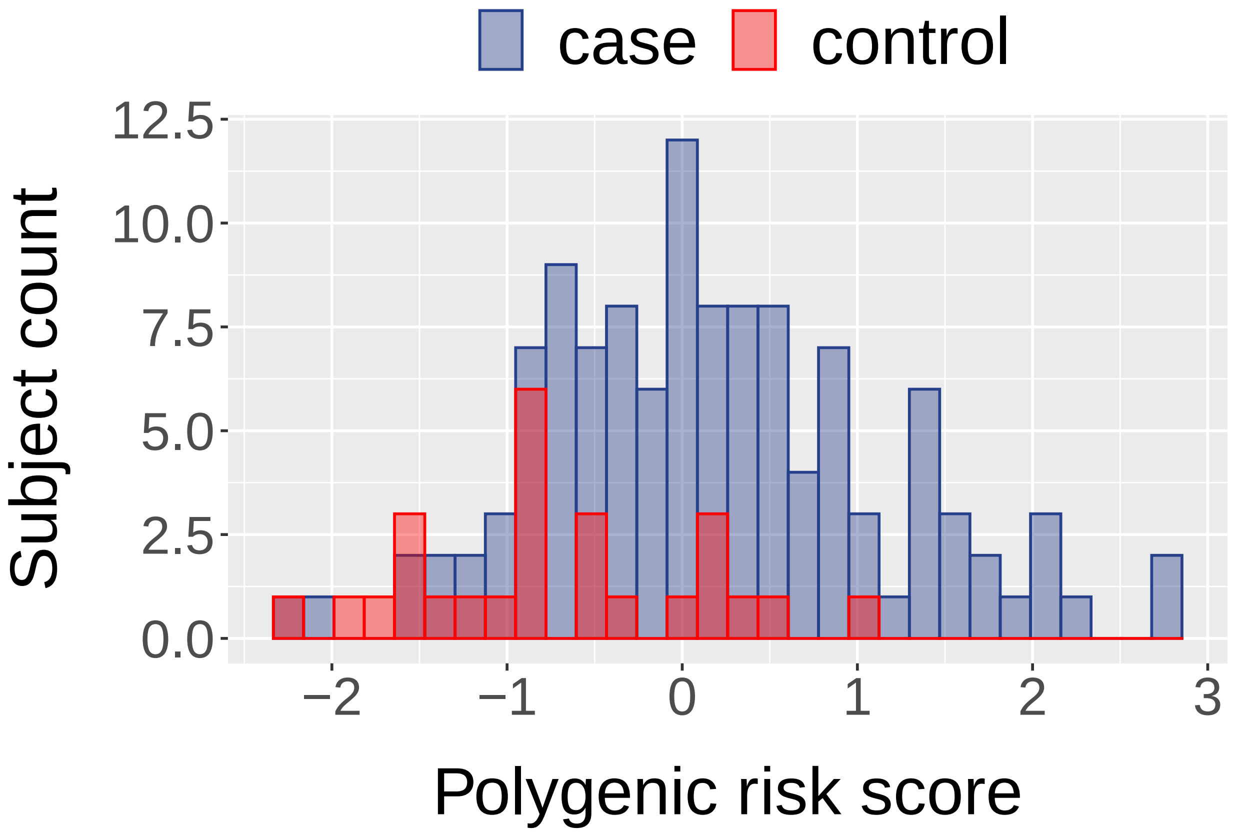


```
##########################################################
# boxplot of genetic risk score/polygenic risk score
##########################################################
grs_R <- read_excel("H:/CHG/Shukla_Sanjay/Noha/MS Gut Microbiome/16s manuscript with genetic risk score/
ggplot(grs_R, aes(x = reorder(Cluster, GRS), y = GRS, fill = Cluster)) +  # Manually specified filling
  geom_boxplot() +
  scale_fill_manual(values = c("royalblue4", "red", "purple")) +
  theme(text = element_text(size=20)) +
  geom_jitter(shape=16, position=position_jitter(0.2)) +
  theme(legend.title = element_text(size=20)) +
  theme(legend.text = element_text(size=15))+
  ylab("Polygenic risk score")+
  xlab("Cluster")
```

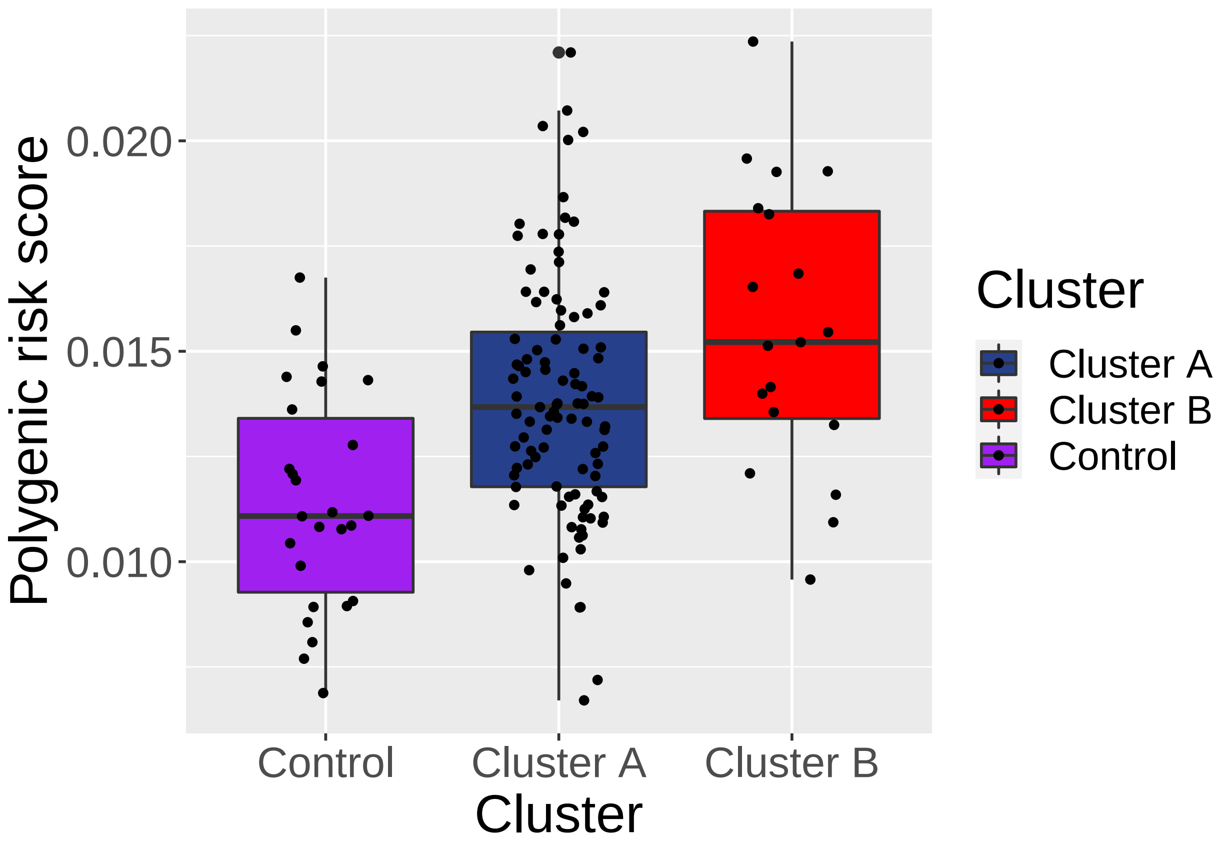

Polygenic risk score
0.020
0.015
0.010
Control
Cluster A
Cluster B
Cluster
Cluster
Cluster A
Cluster B
Control
